# Molecular mechanisms of tigecycline-resistance among *Enterobacterales*

**DOI:** 10.3389/fcimb.2024.1289396

**Published:** 2024-04-09

**Authors:** Lukasz Korczak, Piotr Majewski, Dominika Iwaniuk, Pawel Sacha, Mariola Matulewicz, Piotr Wieczorek, Paulina Majewska, Anna Wieczorek, Piotr Radziwon, Elzbieta Tryniszewska

**Affiliations:** ^1^ Department of Microbiological Diagnostics and Infectious Immunology, Medical University of Bialystok, Bialystok, Poland; ^2^ Regional Centre for Transfusion Medicine, Bialystok, Poland

**Keywords:** tigecycline, glycylcyclines, efflux pumps, multidrug resistance (MDR), *Enterobacterales*

## Abstract

The global emergence of antimicrobial resistance to multiple antibiotics has recently become a significant concern. Gram-negative bacteria, known for their ability to acquire mobile genetic elements such as plasmids, represent one of the most hazardous microorganisms. This phenomenon poses a serious threat to public health. Notably, the significance of tigecycline, a member of the antibiotic group glycylcyclines and derivative of tetracyclines has increased. Tigecycline is one of the last-resort antimicrobial drugs used to treat complicated infections caused by multidrug-resistant (MDR) bacteria, extensively drug-resistant (XDR) bacteria or even pan-drug-resistant (PDR) bacteria. The primary mechanisms of tigecycline resistance include efflux pumps’ overexpression, *tet* genes and outer membrane porins. Efflux pumps are crucial in conferring multi-drug resistance by expelling antibiotics (such as tigecycline by direct expelling) and decreasing their concentration to sub-toxic levels. This review discusses the problem of tigecycline resistance, and provides important information for understanding the existing molecular mechanisms of tigecycline resistance in *Enterobacterales*. The emergence and spread of pathogens resistant to last-resort therapeutic options stands as a major global healthcare concern, especially when microorganisms are already resistant to carbapenems and/or colistin.

## Enterobacterales

The order *Enterobacterales* comprises a relatively large group of gram-negative microorganisms associated with both human healthcare and agricultural settings. *Enterobacterales* consists of many different species, the vast majority causing opportunistic infections for example via transmission of strains from the human gut to the urinary tract. Previously recognized as relatively harmless, they are now known as causing nosocomial infections with high levels of antibiotic resistance or even pan-drug resistance (PDR) (Howard et al., 2012). Nosocomial infections caused by drug-resistant *Enterobacterales* are especially harmful to patients with chronic diseases, immunocompromised patients and even to healthcare workers. Common risk factors for these types of infections include previous treatment with broad-spectrum antibiotics, extended hospitalization time, admission to the intensive care unit (ICU), defective immune system, controlled mechanical ventilation, transplantations, congenital heart disease, coronary artery bypass grafts and advanced age (Bazaid et al., 2022; Deng Y. et al., 2022). Specifically, the presence of multi-drug resistant (MDR) pathogens raises a noteworthy concern. MDR strains often occurring in intensive care units have shown an alarming mortality rate due to the difficulty of treatment with antibiotics (Wang P. et al., 2022). According to susceptibility levels in the Asia-Western Pacific region, tigecycline treatment is currently effective in most MDR infections (Li et al., 2015). However, bacterial resistance to last-line agents is increasingly reported worldwide, probably due to antibiotic misuse or overuse. The economic costs of hospital treatment of MDR infections and their mortality rate are also growing alarmingly (Silva et al., 2019).

## Tigecycline

Tetracyclines are known for their broad spectrum of activity against microorganisms including a wide range of gram-positive and gram-negative pathogens. The first tetracyclines were obtained from *Streptomyces aureofaciens* in 1948 and approved by the U.S. Food and Drug Administration (FDA) for clinical use the same year (Grossman, 2016). The growing development of resistance to many classes of antimicrobial agents forced scientists to search for new agents that can cope with the increasing prevalence of MDR or extensively drug-resistant (XDR) pathogens worldwide.

**Figure 1:**
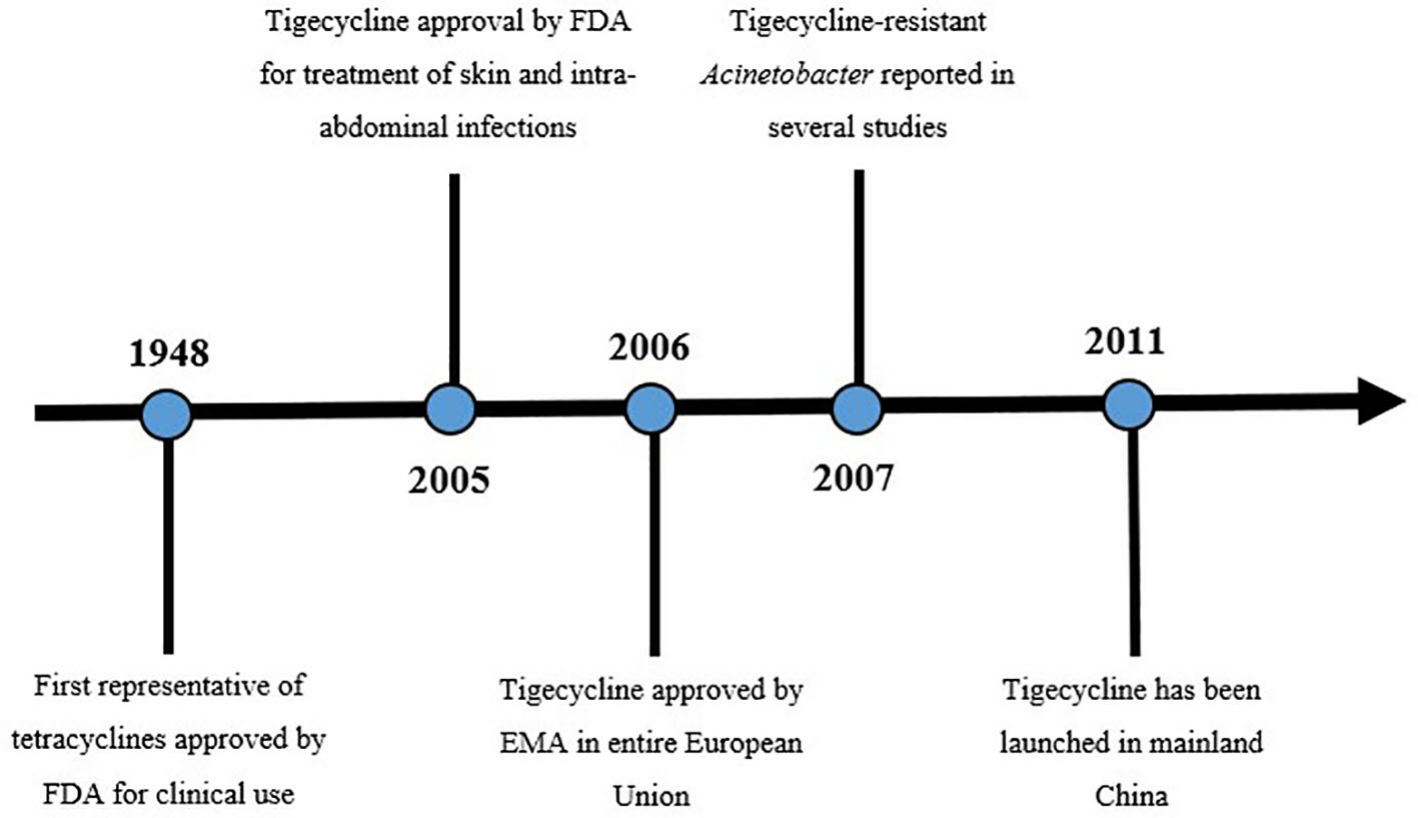
Timeline of tigecycline (Yaghoubi et al., 2022). Figure exhibits a timeline from first approval of tetracycline to the launch of tigecycline in China. Before tigecycline use in China there were reported resistant *Acinetobacter* strains.

Tigecycline is the first representative of a new class - glycylcyclines. It is a chemically modified minocycline (a 9-t-butyl glycol amide derivative of minocycline) with a molar mass of 585 g/mol (Yaghoubi et al., 2022). Tigecycline was approved by the FDA in 2005 for the treatment of complicated skin and intra-abdominal infections. The European Medicines Agency (EMA) approved tigecycline in 2006, and 2011 it was launched in China. Interestingly, six strains of *Klebsiella pneumoniae* that were resistant to tigecycline before 2011 were discovered in China (Zhong et al., 2014). Moreover, clinical resistance to tigecycline has been reported worldwide since 2007. Interestingly, before tigecycline use in China there were reported resistant Acinetobacter strains. (**Figure 1**) Yuhan and colleagues observed that the tigecycline resistance rate increases year to year (Yuhan et al., 2016). There were many reports indicating tigecycline resistance in various pathogens, such as *Klebsiella*, *Eschericia coli*, *Enterobacter* spp. and other *Enterobacterales* (especially enzyme producing). The percentage of non-susceptible strains ranged from a few percentages to almost 80% (Sun et al., 2013; Yuhan et al., 2016). However, a study by Zhong and colleagues indicated that reported tigecycline resistance could be indirectly attributed to the previous use of other antibiotics, previously transported by the same efflux pump (Zhong et al., 2014). In present times, tigecycline is approved for the treatment of complicated skin and skin-structure infections, complicated intra-abdominal infections and community-acquired pneumonia, as a monotherapy (Hung et al., 2009). Tigecycline is also lately recognized as one of the last-resort antimicrobial agents against MDR/XDR/PDR *Enterobacterales* (Yu Y. et al., 2022).

The mechanism of tigecycline action is comparable to tetracycline-embracing inhibition of bacterial-protein translation. The whole process is possible by binding to the bacterial ribosome 30S subunit. Disruption of peptide synthesis and bacterial metabolism results in bacteriostatic properties of this antimicrobial agent. Tigecycline is a parenteral antibiotic, compiled to overcome the main mechanisms of resistance to tetracyclines, for instance, ribosomal protection, active drug efflux or enzymatic deactivation (Pankey, 2005; Jenner et al., 2013; He et al., 2018).

Tigecycline has a broad antimicrobial spectrum including gram-positive (*Staphylococcus aureus* and methicillin-resistant *S. aureus* strains, *Streptococcus pneumoniae*, *Streptococcus agalactiae*, *Enterococcus* spp. including vancomycin-resistant strains), gram-negative (*Enterobacterales* - *Klebsiella pneumoniae*, *Enterobacter cloacae*, *Serratia marcescens*, extended-spectrum beta-lactamase (ESBL) producing strains and carbapenem-resistant (CRE strains), anaerobes, and atypical microorganisms. Some pathogens like *Morganella* spp., *Proteus* spp., *Providencia* spp., and *Pseudomonas aeruginosa* exhibit natural resistance to tigecycline.

## Development of MDR/XDR strains

Today, at the edge of the post-antibiotic era, tigecycline and colistin are considering the most effective against MDR and XDR strains, posing the last-chance treatment option for several infections (Ojdana et al., 2015; Majewski et al., 2020; Kwon and Powderly, 2021). A crucial example is CRE, spreading worldwide and constantly reducing available therapeutic options. The presence of CRE was detected on five continents - Europe, Asia, Africa, and North and South America. Notably, almost 50% of CRE strains displayed tigecycline resistance (Lucaßen et al., 2021). It is worth noting that most CRE strains are not only resistant to carbapenems but also resistant to at least one drug among other antimicrobials, subsequently leading to XDR or even PDR development (Yuhan et al., 2016). Colistin seems to be a poor choice for patients due to severe side effects including nephrotoxicity, neurotoxicity (paresthesia, partial deafness, confusion, hallucinations, seizures, ataxia), and electrolyte imbalance. Significant side effects associated with colistin force physicians to treat infection caused by MDR pathogens using tigecycline (Ordooei Javan et al., 2015; Aksoy et al., 2020). Adverse drug reactions of tigecycline are mild, including gastrointestinal problems (diarrhoea, nausea, vomiting), pancreatitis, fever, accompanied coinfections, thrombocytopenia, local reaction, and changes in hepatic functions (increased concentration of alanine transaminase and aspartate transaminase) (Greer, 2006; Tasina et al., 2011).

According to a European Centre for Disease Prevention and Control report, the amount of carbapenem-resistant *Enterobacterales* is still increasing, especially in central and eastern Europe, Spain and Italy (> 50% of invasive isolates resistant to imipenem/meropenem). In western Europe and Scandinavia, the number of strains is lower (1-5%). Interestingly, carbapenem-resistant *E. coli* strains pose <1% of all isolates (European Centre for Disease Prevention and Control, 2019).

**Figure 2:**
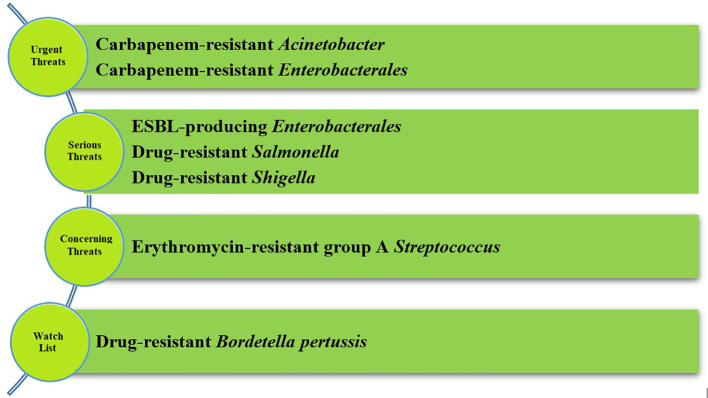
Groups of hazards according to CDC report (the most crucial microorganisms) (CDC, 2019). CDC divided pathogens in the four groups of hazards. In serious threats and urgent threats there are *Enterobacterales* and their representatives, primarily carbapenem-resistant *Enterobacterales*.

In 2019 the CDC published a report concerning antibiotic resistance in the United States, where four groups of microorganisms were distinguished and categorized into four groups of hazards (the most important pathogens are presented in **Figure 2**). The first group contained urgent threats to human health. Carbapenem-resistant *Acinetobacter* and carbapenem-resistant *Enterobacterales* are representatives of this group. In serious threats, there are drug-resistant *Salmonella* and *Shigella* strains and ESBL-producing *Enterobacteriaceae*. Other pathogens in the CDC report are gram-positive and gram-negative bacteria (CDC, 2019).

## Mechanisms of tigecycline-resistance

The incidence of tigecycline resistance has been increasing yearly since 2007 (Sun et al., 2013). In recent decades, many strains of tigecycline-resistant pathogens have been reported in France, Germany, Greece, Saudi Arabia, Spain, Taiwan, the United Kingdom and the United States (Goodarzi et al., 2021). Interestingly, a tigecycline-resistant *Acinetobacter baumannii* strain was isolated in Iran, but tigecycline was not approved in this country at that time (Goodarzi et al., 2021).

Different mechanisms of tigecycline resistance and resistance to related antimicrobial agents (like tetracyclines) are conferred by efflux pumps, (such as AcrAB-TolC); enzymatic inactivation (via *tet*(X) genes) ribosomal-protection proteins (thus proteins encoded by *tet*(M) genes) (Sun et al., 2018); and pump regulators like AcrR, MarA, SoxS, RamA, RarA (Zhong et al., 2014).

During recent years, tigecycline resistance has emerged and has mostly been identified in gram-negative bacteria, mainly *Enterobacterales* and *Acinetobacter* spp (Jean et al., 2022).

**Figure 3:**
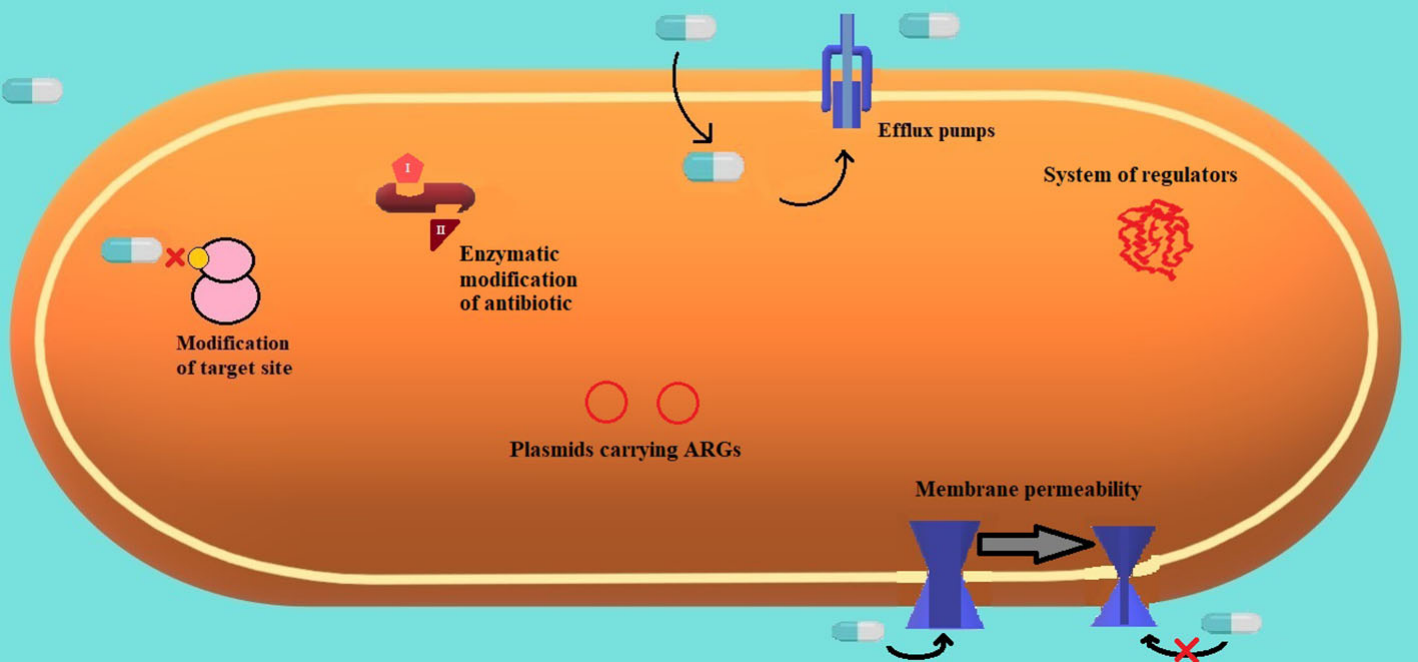
Frequent mechanisms of resistance are present in gram-negative strains (Sharma et al., 2019).

Interestingly, a phenomenon of heteroresistance may develop during infections treated with tigecycline (mechanisms are presented in **Figure 3**) (Wang Q. et al., 2022). Tigecycline resistance can either be intrinsic or acquired via horizontal gene transfer (HGT), making these mechanisms suitable for heteroresistance. Heteroresistance is defined as the presence of a subpopulation of cells with higher minimal inhibitory concentration (MIC) than the dominant population existing in one sample (El-Halfawy and Valvano, 2015; Stojowska-Swędrzyńska et al., 2021). Moreover, heteroresistance can be an intermediate phase between susceptibility and resistance after exposure to antibiotics. More research concerning this phenomenon needs to be completed (Chen et al., 2017).

Genes involved in tigecycline-resistance mechanisms listed below can be encoded chromosomally or located on genetic vehicles, such as plasmids. Some cellular compounds can chop the chromosomal DNA and transfer it to other plasmid or chromosomal sites. Antibiotic resistance genes (ARGs) are recognized as novel hazards, which could be disseminated worldwide simply via various reservoirs such as soil, water, and animal excrement. Certain ARGs can also be a part of a multidrug-resistance region located on a genomic island presented in chromosomal DNA (Li et al., 2022). Moreover, a phenomenon of the coexistence of several resistant genes was reported and previously described in *Enterobacterales* (Wang Q. et al., 2022). Genes encoding resistance to tigecycline and resembling agents, such as *tet*(X), can coexist with various carbapenemases and *mcr* variants, causing resistance to carbapenems and colistin (Wang Q. et al., 2022). Coexistence of genes on a single plasmid may affect wide dissemination and persistence under different selective pressure, which can explain its presence in human and animal clinical specimens (Wang J. et al., 2020). Plasmids play a crucial role in carrying multiple functional genes and transferring them into bacteria through conjugation (Wang Q. et al., 2022). Furthermore, ARGs show a major role in the diversification and genomic plasticity of bacteria by HGT (Li Y. et al., 2021). Interestingly, the presence of the AcrAB efflux pump upon exposure to antimicrobial agents, e.g., ciprofloxacin, can result in cross-resistance to tigecycline. This situation is highly alarming, as it may lead to the potential emergence and selection of additional mechanisms of cross-resistance to novel antimicrobial agents in the future (Chen et al., 2017).

Frequent mechanisms of resistance are present in gram-negative strains (Sharma et al., 2019). (**Figure 3**.) The most common mechanisms of resistance embrace modification of the target site and antibiotics’ inability to bind, enzymatic modification of an antibiotic to a harmless compound, efflux pumps extruding toxic substances (like antibiotics), plasmids carrying genes responsible for antimicrobial resistance, and variable membrane permeability and systems of versatile molecular regulators.

## Efflux pumps

Efflux pumps are proteins involved in the extrusion of substrates from the bacterial cell to the environment (Sharma et al., 2019). Bacterial efflux pumps are capable of expelling a wide variety of substances like bacterial metabolites, antimicrobial agents, detergents, metabolites, dyes, toxins, virulence factors, heavy metals, organic pollutants, plant-produced compounds, quorum-sensing signals, antiseptics, disinfectants, and preservatives (Marquez, 2005; Blanco et al., 2016; Alenazy, 2022). Moreover, these pumps actively contribute to the biofilm formation. It has been proved that pumps belonging to the major facilitator superfamily (MFS) and resistance-nodulation division (RND) families play a significant role in the process of biofilm development in *E. coli* (Alav et al., 2018). Furthermore, the overexpression of AdeFGH efflux pump in clinical strains of *A. baumannii* was correlated with biofilm formation (He et al., 2015). Interestingly, overexpression of AdeABC and AdeIJK may result in the reduction of biofilm formation, due to a reduced expression of certain genes encoding proteins crucial to the entire process (Richmond et al., 2016). The mechanics of these structures are energy dependent to facilitate the transport of various substances against a concentration gradient (Sharma et al., 2019). Genes coding efflux pumps are found mainly in the bacterial chromosome (Blanco et al., 2016). Additionally, pumps can be divided into two groups: substrate-specific pumps and pumps working with a broad spectrum of various compounds. Interestingly, the second group is usually present in MDR pathogens.

Efflux pumps can differ among five superfamilies, based on their properties, such as topology in the membrane or sources of energy (Du D. et al., 2018): ATP-binding-cassette (ABC), multidrug and toxin extrusion (MATE), MFS, small multidrug resistance (SMR), and RND. The last group is present strictly in gram-negative microorganisms (AlMatar et al., 2021). Development of tigecycline resistance is frequently related to overexpression of efflux pumps belonging to the RND family. It is proven, that RND is considered to be the main efflux pump family, as it confers a multidrug resistance to various gram-negative microorganisms (Alenazy, 2022). RND pumps are chromosomally encoded, consisting of three parts – inner membrane-spanning pump subunit, outer membrane pore, and linker protein. Efflux pumps seem to be one of the most effective mechanisms that confer MDR phenotype to different types of bacteria. It prevents an accumulation of antimicrobial drugs inside the cell, reducing the concentration of antibiotics to non-toxic levels. Overexpression of AcrAB and OqxAB pumps, representatives of the RND superfamily, is the most frequent mechanism reported in *Enterobacterales (* He et al., 2018 *).* Moreover, it is thought that other efflux pumps from the RND superfamily, such as AdeABC, AdeFGH, and AdeIJK, are confirmed to mediate in tigecycline resistance in *A. baumannii* strains (Chen et al., 2014). Furthermore, AdeABC and AdeIJK efflux pumps are recognized as other contributors to tigecycline resistance (Lucaßen et al., 2021).

Regulations of MDR efflux pumps are similar in different species; there is a local repression of pump genes as well as global transcription-factor regulation. For instance, AcrR works as a repressor to prevent overexpression of *acrAB*; *acrR* is based upstream of the *acrAB* operon, and as a result, can repress its synthesis (Weston et al., 2018). The best-characterized RND pump is AcrAB-TolC and it is widely present in *Enterobacterales* (*E. coli*, *Salmonella*). In 2004, a new plasmid-encoded efflux pump, OqxAB was reported on a plasmid located in *E. coli* isolated from pig faeces in Denmark (Hansen et al., 2004). Since 2004, the new efflux pump has been investigated widely. The prevalence of *oqxAB* in *Enterobacterales* has been increasing over the past decades. Scientists proved that overexpression of OqxAB confers resistance to antimicrobial agents (chloramphenicol, quinolones, nitrofurantoin, tigecycline), as well as detergents and disinfectants (Li et al., 2019). Interestingly, decreased susceptibility to tigecycline is often associated with overexpression of efflux pumps from the RND superfamily, like AcrAB-TolC, OqxAB, and AdeABC. Mutations in *acrR* may cause a loss in repressor function, resulting in *acrAB* overexpression (Chen et al., 2017).

**Figure 4:**
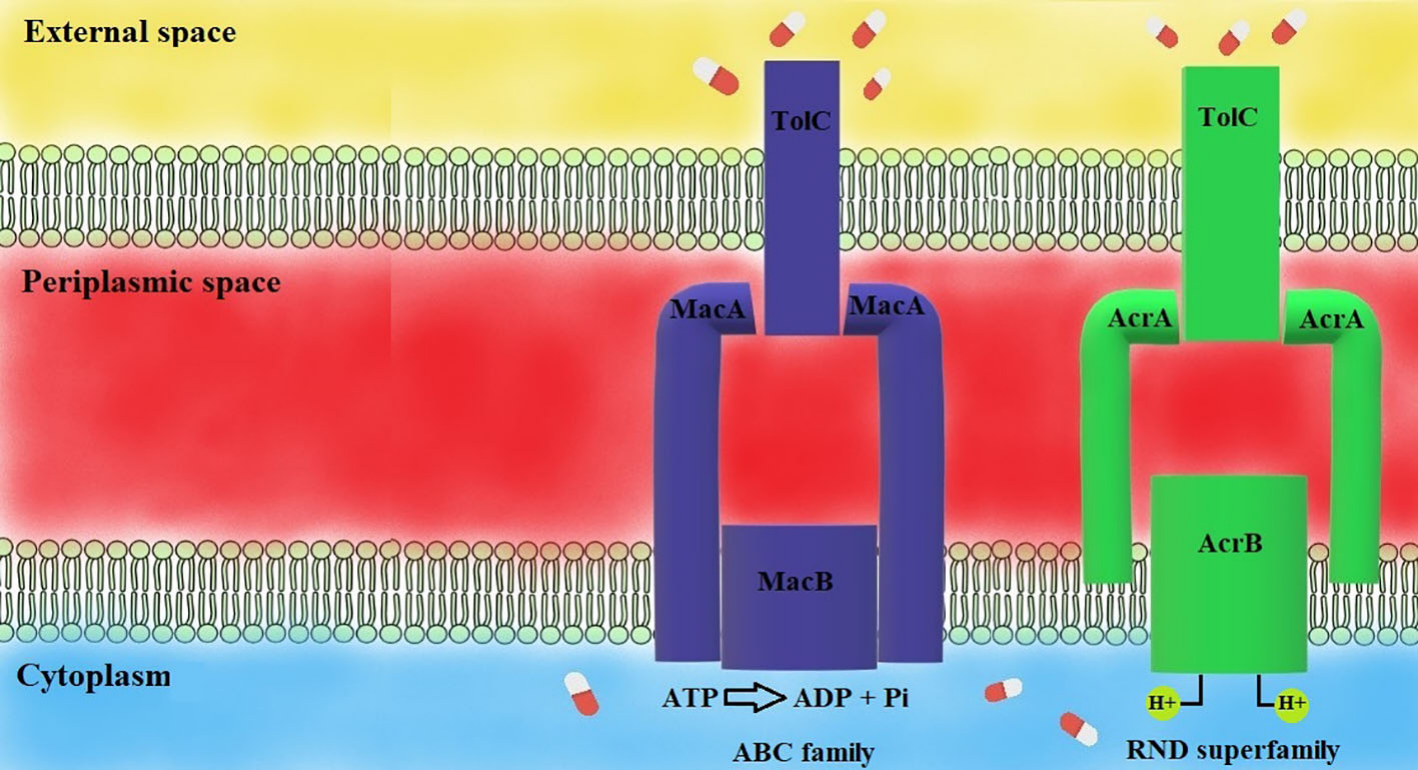
Mechanisms of efflux pumps (Sharma et al., 2019). MacAB-TolC pump consists of three elements: MacB and TolC, which extrude toxic substances, the MacA-MacB linkage and TolC. MacAB-TolC is an ATP-dependent pump. AcrAB-TolC pump is a representative of the RND family, consisting of three parts: AcrB and TolC, extruding harmful substances, AcrA, which links AcrB, and TolC. AcrAB-TolC efflux pump is a proton-motive force-dependent efflux pump.

AcrAB-TolC efflux pump comprises three components: AcrB is responsible for substrate recognition and utilizing the proton motive force; TolC excretes substrates from bacterial cells; and AcrA connects TolC with AcrB, and prevents failure of transport across the periplasm. Inhibition of one component can inhibit the entire efflux pump (pumps shown in **Figure 4**) (Nikaido and Takatsuka, 2009). AcrB is the most well-characterized member of the RND superfamily. High expression of AcrAB-TolC and OqxAB efflux pumps plays a crucial role in tigecycline resistance (Goodarzi et al., 2021).

The AcrB transporter undergoes a cyclic process involving three structural states: access, binding, and extrusion. Drugs enter the binding pocket during the access state, move to the binding state and are expelled in the extrusion state. The passage-through states is linked with the protonation of side chains. Two five-helix repeats (R1 and R2) and flanking helices are crucial for these transitions. The protonation changes contribute to the cooperative drug binding and extruding by the AcrB transporter (Murakami et al., 2002; Du D. et al., 2018). Studies based on X-ray crystallography and cryo-electron microscopy revealed different drug pathways in the AcrB protein. There are various pathways involving the proximal binding pocket (PBP) in the L promoter, transporting drugs to the distal binding pocket (DBP). Different drugs are specific to various pockets depending on their molecular mass. Drugs with a higher mass like erythromycin and rifampicin can access a pocket in the L monomer. Smaller drugs like minocycline can access a binding pocket in the T monomer (Murakami et al., 2002; Nakashima et al., 2011; Du D. et al., 2018). Due to the fact, that tigecycline is a derivative of minocycline, it is suspected that tigecycline acts in a way that is similar to minocycline.

The MacAB-TolC efflux pump is a well-known representative of the ABC family. It consists of three components: MacB, MacA, and TolC. MacA connects TolC in the outer membrane and MacB in the inner membrane. There is a structure discovered in the MacA component working as a gating ring – it acts like a valve providing one-way substrate transport. MacAB-TolC is an ATP-dependent efflux pump. MacA and MacB components are responsible for hydrolyzing ATP with ATPase enzyme. The MacAB-TolC efflux pump is responsible for the active extruding of substrates, including virulence factors (like heat-stable enterotoxin) and antibiotics (mainly macrolides) (Yamanaka et al., 2008; Fitzpatrick et al., 2017). A study performed by Lin and colleagues revealed that there is increased expression of genes coding components of the MacAB-TolC efflux pump upon exposure to tigecycline *in vitro* (Lin et al., 2015). This phenomenon suggests a possibility of extruding tigecycline out of the bacterial cell by the discussed efflux pump.

## The history and origin of the *tet*-genes family

In recent years tigecycline resistance in MDR bacteria has been associated with overexpression of efflux pumps or mechanisms of ribosomal protection. The prevalence of the novel mobile *tet* family is one of the essential mechanisms of resistance to tetracyclines and glycylcyclines. Furthermore, *tet*(X) activity renders first-line antimicrobial agents (such as tetracyclines) ineffective. *Tet* genes (the most known representative is the *tet*(X) gene) encode functional proteins, working as enzymes. *Tet*(X) is flavin-dependent monooxygenase (FMO) consisting of 388 amino acids, capable of catalyzing hydroxylation of tetracyclines to 11α-hydroxy-tetracyclines, making it inefficient (Cui et al., 2021). Phylogenetic analysis performed by Zhang and colleagues showed that *tet*(X) genes remain stable in various environments (they are stable in various regions and countries). Moreover, *tet*(X) genes can differ into two clusters based on their origin and similarity; one cluster contains *tet*(X), *tet*(X2), *tet*(X3), and *tet*(X4) genes, and the second cluster contains the *tet*(X1) gene, which included the highest diversity. Even though *tet*(X) genes can differ in different phylogenetic lines, they remain functionally similar (Cheng et al., 2022). In 1984, a transferable *tet*(X) gene was discovered on a plasmid isolated from *Bacteroides fragilis* originating from human samples. This phenomenon was the earliest prevalence of antibiotic resistance genes that could inactivate tetracyclines (and glycylcyclines subsequently) (Ghosh et al., 2009). According to current reports, *tet*(X) genes occurred in *Riemerella anatipestifer* in 1966, collected from ducks in the United Kingdom (Zhang et al., 2021). Additionally, tigecycline was identified as a *tet*(X) substrate in 2005 (the same year the antibiotic was approved by the FDA). Interestingly, the *tet*(X) gene-encoded enzyme is effective only under aerobic conditions (it’s worth noting, that *Bacteroides* are anaerobic bacteria). Except for oxygen, other required substances are flavin adenine dinucleotide (FAD), NADPH and magnesium ions (Moore et al., 2005). Today, bacterial *tet* genes have spread to many different regions on five continents (Africa, Asia, Europe, and North and South America) (Fang et al., 2020). According to a recently published report, the occurrence of *tet*(X) is highest in Spain (9,4%), Germany (5,1%), France (3,3%), Denmark (3,2%) and China (3,2%) (Zhang et al., 2021). *Tet*(X) variants, *tet*(X1) and *tet*(X2) were originally isolated from *Bacteroides* transposon, and had 61,7% and 99,5% amino acid sequence similarity, respectively. *Tet*(X2) caused resistance to antimicrobial agents, but *tet*(X1) was inactivated, due to incomplete gene structure. Moreover, *tet*(X1) and *tet*(X2) genes exhibited a low level of resistance to glycylcyclines (MIC ≤ 2µg/ml) (Fang et al., 2020). Interestingly, it has been noticed that *tet*(X) become widely disseminated among different species of bacteria, for example, *E. coli* strains (Zhang et al., 2022). A study performed by Sun and colleagues revealed that plasmids from Inc families can also carry *tet*(X) genes. Thus far, plasmid families harboring the *tet*(X) genes include IncF, IncN, and IncQ. IncQ plasmids are relatively small plasmids. The PLHM10-1-p6 plasmid present in *E. coli* strains can be transferable, but a helper plasmid is needed. The presence of a helper plasmid is essential for the mobilization of plasmids carrying *tet*(X4) genes. Notably *mcr-1* plasmids can act as helper plasmids, potentially enhancing the chance of dissemination of plasmid-mediated tigecycline resistance (Sun J. et al., 2019). Various studies revealed the spread of genes through HGT (Wang P. et al., 2022). *Tet* genes can be found in plasmids harboring IncQ1. Moreover, these plasmids are recognized as highly transferable. A study performed by Sun et al. revealed that the pLHM10-1-p6 plasmid originates by the acquisition of the *tet*(X4) gene. *Tet* variants are widely present in many bacterial species including *Enterobacterales*, such as *E. coli*, *Salmonella*, *Proteus*, and *Providencia* (Zhang et al., 2022). Furthermore, *tet* genes were observed in both environmental bacteria and MDR human pathogens. Interestingly, *tet*(X) occurs in different reservoirs, such as humans, wild animals, agriculture, and livestock. Moreover, several *tet*(X) gene variants were primarily isolated from animals. Some of them (like the *tet*(X4) gene) are widely disseminated in food-producing animals, meat for human consumption, migratory birds, shrimp meat, humans, and environmental samples, *i.e*., soil, river water, and environmental aerosols (Sun C. et al., 2019; Mohsin et al., 2021). In most geographic regions antimicrobial agents are widely overused in many areas of life and industry. The vast majority of antimicrobial pollutants occur in the environment and stand as the main driving force of ARG dissemination. Even in Europe, despite the fact that the European Union prohibited the usage of antibiotics as growth promoters, there were cases of tetracycline-resistant *E. coli* in food-producing animals (Zhang et al., 2015; Sun J. et al., 2019). Moreover, tigecycline is the only drug approved for clinical use and it was never considered withdrawn in the veterinary field (Fang et al., 2020). In recent years many more *tet* orthologs were isolated, such as *tet*(X5) – *tet*(X14). These genes were first obtained in food animals – *tet*(X3), *tet*(X4), *tet*(X6) and *tet*(X14) (Zhang et al., 2021). As previously mentioned, many *tet*(X*)* variants were isolated through the years. In 2001, *tet*(X1) and *tet*(X2) genes were found. *Tet*(X1) is unable to degrade tetracyclines and glycylcyclines due to a lack of FAD-binding domain, but *tet*(X1) and *tet*(X2) can cause low resistance to tigecycline (Moore et al., 2005). Additionally, *tet*(X3) and *tet*(X4) were identified in *Acinetobacter* and *Escherichia* strains with 85% and 94% amino acid identity to original *tet*(X), respectively. These two orthologs confer high-level tigecycline resistance (<16 mg/l) and are considered a major threat to global health.

## Transfer of *tet* genes

Interestingly, the transfer of mentioned *tet*(X) orthologs was found to be plasmid-mediated (He et al., 2019). *Tet*(X3) and *tet*(X4) were recently proven to occur in various environments. This phenomenon is caused by the transfer of these genes on mobile elements and becomes a serious threat, especially in hospital settings (Li Y. et al., 2021). Another interesting fact is that tigecycline was never used in animal husbandry in China, but mobile tigecycline resistance genes, *tet*(X3) and *tet*(X4), were discovered in strains isolated from pigs (Wang Y. et al., 2020). This situation proves that transfer on mobile elements, such as plasmids or transposons, is a decisive threat and can occur in other environments. The study performed by Liu and colleagues showed that *tet* genes can be detected in bacterial genomes and, notably, their horizontal transfer is not limited to plasmids (Liu D. et al., 2020). Scientists isolated two *tet*(X2) genes and a *tet*-like gene, whose size was 1137 bp and whose identity was 87,8%, 87,6%, 79,4%, 90,2%, and 92,1% similar to *tet*(X), *tet*(X2), *tet*(X3), *tet*(X4), and *tet*(X5), respectively (Liu D. et al., 2020). Genomic analysis showed, that similar proteins were present in genomes of *E. coli*, *Acinetobacter* and *Pseudomonas* strains isolated from pigs and chickens. Recently, the novel *tet*-like gene was described as *tet*(X6) (Liu D. et al., 2020). Furthermore, the novel *tet*(X6) gene exhibited a lower level of resistance to glycylcyclines than other variants of genes (Liu D. et al., 2020). A novel *tet*(X15) gene was isolated from *Acinetobacter* strains. Genomic analysis proved that *tet*(X15) was located on chromosomal transposons. Moreover, the new *tet*(X15) gene demonstrated about 91% to 96% similarity to other *tet*(X) variants. Also, *tet*(X15) conferred resistance to tetracyclines and glycylcyclines (Li R. et al., 2021). New variants of the *tet* gene are still emerging, with twenty-seven variants isolated in 2021 (Yu R. et al., 2022). These variants were named from *tet*(X18) to *tet*(X44) and were obtained from *R. anatipestifer* (Umar et al., 2021). *Tet*(X) genes can coexist with other antimicrobial resistance genes, such as *bla*_NDM_ and *mcr*, conferring resistance to carbapenems and colistin (Wang Q. et al., 2022). This particular phenomenon poses a significant challenge for clinicians, as the coexistence of *tet*(X) genes and *mcr* genes can lead to resistance against last-resort antimicrobial drugs such as glycylcyclines and polymyxins. Yongchang and colleagues have recently reported an *E. coli* strain carrying a single plasmid containing both *tet*(x) genes and the *mcr-1* gene (Xu Y. et al., 2021). Another interesting fact is, that *tet*(X) derivatives confer MIC for tigecycline in a way that is similar to MIC in the presence of AcrAB. Moreover, there were observed strains with *ramA* overexpression and *tet*(X) carriage. The situation is rather uncommon, due to the rare occurrence of *mcr-1* genes with other resistance genes on a single plasmid (Xu Y. et al., 2021). Additionally, sporadic dissemination of mobile elements between microorganisms can result in widespread resistance to these last-chance antibiotics. *Tet*(X4) and *cfr* genes were also present on the same plasmid in *E. coli* strains (Tang et al., 2021). Another study demonstrated the presence of *tet*(X6) and *cfr* genes on a plasmid within a *Proteus* strain (Peng et al., 2020). Moreover those genes exhibited high transferability (Tang et al., 2021). Potential dissemination could be a crucial threat for clinicians who treat infections caused by pathogens carrying those plasmids.

## Other *tet* genes and future perspectives

*Tet*(M) was isolated in 2004 in *E. coli* strains obtained from breeding animals, and fully described in 2017 in *S. enterica* obtained from animal faeces in China (Jurado-Rabadán et al., 2014; Liu YY. et al., 2020). Tet(M) is a ribosome protective protein, preventing tetracyclines from binding to the 23S rRNA site and demonstrating a lack of antimicrobial effect (Yu et al., 2021). The presence of the *tet*(M) gene in *E. coli* causes tetracycline, doxycycline, and minocycline resistance. Due to the similarity of minocycline and glycylcyclines, Tet(M) protein has the potential to provide mutation leading to increasing MIC of tigecycline (Sun et al., 2018). The expected mechanism is the interaction between amino acid sequences of Tet(M) which confer resistance to glycylcyclines (Yu et al., 2021). Possible dissemination of this gene can lead to failure of tetracycline- and tigecycline therapy (Sun et al., 2018).

Other variants of *tet* genes were found, including *tet*(B) and *tet*(A). In 2018 these properties were first reported during tigecycline treatment (Du X. et al., 2018). Firstly, in 2016 tigecycline resistance *in vitro* caused by *tet*(A) was reported in *E. coli* strains. Tet(A) works as an efflux pump belonging to the MFS family and its mutation can lead to the accumulation of tigecycline in bacterial cells and result in glycylcycline resistance (Linkevicius et al., 2016). Mutants bearing *Tet*(A) can increase tigecycline’s MIC two- to four-fold in comparison to wild types. Furthermore, a study performed by Chiu et al. revealed, that mutations in *ramR* and *tet*(A) lead to an increase of tigecycline’s MIC. Strains containing type 1 or type 2 mutated *tet*(A) exhibited an eight-fold and four-fold increase in MIC values, respectively. Strains containing a deletion in *ramR* exhibited significantly higher MIC values, indicating a synergistic mechanism of tigecycline resistance (Chiu et al., 2017). Genes located on different plasmids may lead to different expression levels of *tet*(A) and various resistance to antibiotics (Xu J. et al., 2021). Furthermore, *tet*(A) can occur on plasmids coexisting with various resistance genes, such as *bla*_KPC-2_ or *mcr*, providing a risk to widespread carbapenem, colistin, and glycylcycline resistance (Linkevicius et al., 2016).

Tet(B) is a specific transporter of tetracycline and its derivatives. Interestingly, Tet(B) cannot expel tigecycline out of bacterial cytoplasm (Linkevicius et al., 2016). Tet(B) is found in gram-negative pathogens while the Tet(K) transporter is present in gram-positive pathogens, such as *S. aureus* strains. A recent study performed by Hirata and colleagues showed that low concentrations of tigecycline remarkably induced Tet(B). That phenomenon suggests that tigecycline could bind to the TetR repressor and exhibit a higher potential to increase the expression of the Tet(B) efflux pump (Hirata et al., 2004).

Novel *tet* variants have been increasingly reported. One of them is the novel *tet*(Y) gene responsible for tigecycline resistance isolated in *A. baumannii*. Species carrying that gene were sensitive to tigecycline pressure but not susceptible to it, suggesting that tigecycline could cause biochemical changes in bacterial cells. However, tigecycline cannot have a bacteriostatic effect. Moreover, overexpression of *tet*(Y) showed a two- to four-fold increase of tigecycline’s MIC. *tet*(Y) was located on the plasmid and exhibited the potential for transmission via transposable elements (Wang et al., 2021).

To summarize, numerous variants of the *tet* gene family can confer antibiotic resistance in *Enterobacterales*. A summary of *tet*(X) variants and *tet* orthologs is listed in **Tables 1** and **2** respectively. These prevalent variants are frequently identified, especially in MDR strains, posing a significant threat, especially in hospital settings. Multiple studies have reported that the occurrence of *tet* orthologs can result in reduced susceptibility to tigecycline and that the coexistence of other resistance genes can contribute to their dissemination, presenting a serious dilemma for clinicians (Sun et al., 2018).

**Table 1:** The most important *tet*(X) variants.

*Tet*(X) variant	*Tet*(X1)	*Tet*(X2)	*Tet*(X3)	*Tet*(X4)
**Mechanism of action**	Enzymatic degradation of antibiotics	Enzymatic degradation of antibiotics	Enzymatic degradation of antibiotics	Enzymatic degradation of antibiotics
**Possible transmission**	via plasmids	via plasmids	via plasmids	via plasmids
**Coexistence with other resistance genes**	*ermF*, *aadK*,	*ermF*, *aadK*, *floR*,	*bla*_OXA-58_, *bla*_NDM-1_,	*floR, mefB, tetA, sul3*
**Conferring resistance to tigecycline**	(+)	(+)	(+)	(+)
**Reservoir**	Humans, animals,	Humans, animals,	Humans, animals, meat samples,	Humans, animals, meat samples,
**References**	(Zhang et al., 2021)	(Zhang et al., 2021; Zhang et al., 2022)	(Zhang et al., 2020; Zhang et al., 2021)	(Zhang et al., 2020; Zhang et al., 2021; Zhang et al., 2022)

**Table 2:** *Tet* variants – summary.

*Tet* variant	*Tet*(X)	*Tet*(M)	*Tet*(A)	*Tet*(B)
**Mechanism of action**	Enzymatic degradation of antibiotics	Ribosomal protection	Active efflux outside the bacterial cell	Transport protein
**Possible transmission**	via plasmids	via plasmids	via plasmids	via plasmids
**Coexistence with other resistance genes**	*bla*_NDM-1_, *mcr*, *cfr*	Occurrence only with other *tet* variants	*bla*_KPC-2_, mcr	No data
**Conferring resistance to tigecycline**	(+)	(+)	(+)	(+)
**Reservoir**	Animals, humans, environmental samples	Animals, humans, environmental samples	Animals, humans	Animals, humans
**References**	(Wang P. et al., 2022; Zhang et al., 2022)	(Haubert et al., 2016; Sun et al., 2018)	(Xu J. et al., 2021; Yu R. et al., 2022)	(Hirata et al., 2004)

## Other resistance genes

There is a proven existence of several tigecycline resistance genes that do not belong to the *tet* family. These genes are: *plsC*, *trm*, *rpsJ*, *rrf* (ribosome recycling factor), *tviB*, and *msbA*. The most important genes are summarized in **Tables 1**–**3**. All of these genes play a role in conferring tigecycline resistance via various routes. *Trm* genes encode S-adenosyl-L-methionine-dependent methyltransferase, an enzyme protecting the genome of bacteria against unfamiliar DNA and play a crucial role in epigenetic regulations and antibiotic resistance. *Trm* is present in the genome of *Enterobacterales*, catalyzing reactions in the physiological metabolism of bacteria. It participates in the metabolism of proteins, lipids and small molecules. Studies conducted by Chen and colleagues demonstrated that deletion in *trm* leads to a reduction in tigecycline susceptibility (Chen et al., 2014). More investigations need to be done to discover the nature of the *trm* gene and possible therapeutic implications (Chen et al., 2014).

**Table 3:** Other resistance genes – summary.

Name	*msbA*	*rrf*	*gna*	*rpsJ*
**Encoded product**	ABC transporter	protein	polysaccharide enzymes	S10 protein
**Role in bacterial cell**	Transport molecules of lipid A	Dissociate ribosome bound by tigecycline	Encode enzymes taking part in capsule formation	Change the binding site of tigecycline
**Name of bacteria**	*A. baumannii*	*E. coli*	*A. baumannii*	*E. coli*
**References**	(Shilling et al., 2006; King and Sharom, 2012)	(Janosi et al., 1998; Vivanco-Domínguez et al., 2012; Beabout et al., 2015)	(Kenyon and Hall, 2013)	(Hu et al., 2005; Beabout et al., 2015)

*PlsC* genes encode acyl-sn-glycerol-3-phosphate acyltransferase. This enzyme was identified within the cytoplasmic membranes of *E. coli* and it is responsible for catalyzing the biosynthesis of phospholipids. It generally plays a significant role in the biosynthesis of bacterial cell membranes. Moreover, the *plsC* gene is involved in tigecycline insusceptibility towards changes in membrane permeability (Li et al., 2015). Due to those changes, tigecycline cannot cross the bacterial membrane and act properly on the ribosome.

A study performed by He et al. revealed an evolution of the *rpsJ* gene in a patient infected with *Klebsiella*-producing carbapenemases (KPC)-producing *Klebsiella*, which led to tigecycline resistance (He et al., 2018). Initially, the strain was susceptible to tigecycline and the therapeutic impact of tigecycline treatment on the patient was monitored. After twenty-seven days a tigecycline-resistant strain with MIC of 12 mg/l was identified. Whole genome sequencing revealed a single mutation in the *rpsJ* gene. The transformation experiment proved, that mutation in the *rpsJ* gene was responsible for the development of tigecycline resistance. This study provides *in vivo* evidence, that evolution in the *rpsJ* gene can lead to tigecycline resistance among KPC-producing strains during tigecycline therapy. Moreover, the mutated *rpsJ* gene resided on the chromosome of the strain (He et al., 2018).

Other genes, such as *adeS*, *gna*, *rrf*, and *msbA*, are lesser known but also may play a significant role in tigecycline resistance (Hammerstrom et al., 2015). The research performed by Hammerstrom et al. revealed that *adeS*, *gna*, *rrf*, and *msbA* genes may play a role in the adaptation to tigecycline. Moreover, mutations in *msbA* and *rrf* genes exhibited a lower frequency than *adeS* and *gna* genes, suggesting a lesser role in the adaptation of tigecycline. *AdeS* is part of the AdeR-AdeS two-component system. Mutations in the *adeS* gene may cause an AdeABC efflux system overexpression (Lari et al., 2018). Interestingly, the AdeABC efflux pump may be responsible for conferring tigecycline resistance (Hornsey et al., 2010). MsbA is a transporter of ABC, moving a lipid A from the inner membrane to the periplasmic site (Shilling et al., 2006). Moreover overproduction of lipid A can reduce antibiotic transport via MsbA (Woebking et al., 2005). *Rrf* is responsible for dissociating ribosomal subunits (Vivanco-Domínguez et al., 2012). Mutations in this gene may delay the recycling of the TGC-binding ribosomes (Hammerstrom et al., 2015). The *gna* gene is responsible for encoding extracellular polysaccharide biosynthesis enzymes playing a role in capsule formation (Kenyon and Hall, 2013). Mutations of this gene may impact the diffusion of tigecycline into the cell (Hammerstrom et al., 2015). All these genes may lead to tigecycline resistance but more investigations need to be done to determine the contributions of each gene to resistance and their mutual relationships.

## Metals and heavy metals

Metals are widely distributed in the environment. Some of them possess excellent antimicrobial properties. Certain metals can facilitate the development of bacterial resistance to antibiotics. Metal compounds, such as silver and copper compounds can damage proteins, nucleic acids, and other essential structures of bacterial cells (Djoko et al., 2018; Wang et al., 2019).

An analysis performed by He et al. revealed other interesting properties associated with heavy metals (He et al., 2023). Those chemical elements can be present in animal faeces and plant wastes. An analysis showed, that pig faeces, chicken faeces, dairy cow faeces, and plant wastes contained the most crucial metals like lead, mercury, cadmium, chromium, and arsenic. Before 2020 the usage of veterinary tetracyclines as growth promoters contributed to the dissemination of *tet*(X) genes (Zhou et al., 2017). Although China implemented a ban on antimicrobials in animal feed, their impact on *tet*(X) is significant. The ban on antimicrobials has led to an increased significance of heavy metals in the spread of *tet*(X) variants (Sager, 2007; Moral et al., 2009). Moreover metals found in animal faeces are potential carriers of the genes. Heavy metals could act as a consistent selective pressure favoring the spread of *tet*(X) variants. One study confirmed a co-selection effect between heavy metals and *tet*(X) variants and other resistance genes (He et al., 2023). The prolonged use of organic fertilizers (based on animal faeces) can result in the accumulation of metals in the soil or groundwater, creating selective pressure for the future spread of *tet*(X) genes in the environment (He et al., 2023).

Other metals like bismuth and its compounds act differently; for instance, bismuth nitrate, which is used in the treatment of *Helicobacter pylori* infections, peptic ulcers, or diarrhea, can exhibit an interesting impact in terms of antimicrobial susceptibility. Interestingly Deng and colleagues reported that bismuth drugs can restore tigecycline susceptibility in gram-negative strains. The mechanisms are still not clearly explained; however, they can be attributed to biological pathways. Furthermore it appears, that proteins and enzymes (like *tet*(X4)) are probable targets of bismuth drugs (Deng T. et al., 2022). These drugs exhibit an excellent synergistic effect with tigecycline. MIC of tigecycline in strains with the presence of the *tet*(X4) gene was prominently reduced. Moreover, the fractional inhibitory concentration index was synergistic or additive for several bismuth drugs. We can conclude that these drugs can act as adjuvants with tigecycline. An intriguing observation is that bismuth nitrate can inhibit high-level resistance to tigecycline. Bi(NO_3_)_3_ also works as a competitor on the binding sites of Tet(X4) enzymes, preventing the degradation of glycylcyclines (Deng T. et al., 2022).

Heavy metals are another group of molecules, that can be associated with tigecycline resistance. These compounds are part of a group called chemical contaminants, including pesticides. Some heavy metals including As, Cd, Cr, Hg and Pb exhibit a correlation with *tet* family genes. Moreover, heavy metals increase the permeability of the bacterial outer membrane (OM) by damaging its structure. This permeability allows transferable genetic elements, such as plasmids to pass through the OM. The evidence presented above highlights the key role of heavy metals in the dissemination of various *tet* gene variants (Ohore et al., 2019).

## OM porins and membrane permeability

Gram-negative organisms possess bilayer cell walls composed of inner membranes, with a periplasmic space located between the inner membrane (IM) and OM. The OM is a primary line of defense for gram-negative organisms, acting as a mechanical and physiological barrier against possibly harmful external conditions. The OM protects the cell from antibiotics, disinfectants, proteins and other substances. The prevalence, mutual influences, and functionality of OM porins (OMP) is a significant factor in antimicrobial resistance to antimicrobial agents (Majewski et al., 2016). Porins are structures that can facilitate the transport of substances of mass lower than 600 Da (Pagès et al., 2008). Porins pose a vast majority of the total amount of OM proteins in *Enterobacterales*. *E. coli* synthesizes three major porins: OmpF, OmpC and PhoE. The initial two proteins exhibit selectivity toward cationic molecules, whereas the third porin is specific only for anionic molecules. Omp variants are present in *Enterobacter cloacae* and *Enterobacter aerogenes*, the closest members of *Enterobacterales*, and in *Klebsiella* strains (Masi et al., 2017).

Linkevicius et al. observed that interruption in the process of lipopolysaccharide (LPS) biosynthesis may lead to tigecycline resistance (Linkevicius et al., 2013). During the process the “deep-rough” mutant of the LPS structure was observed. Although these condition could be assumed with tigecycline susceptibility, LPS mutants exhibited lower susceptibility (Linkevicius et al., 2013). The mechanism of reduced susceptibility is not certain, although it may be associated with significant reduction of porins in the OM, possibly resulting in insufficient concentration of intracellular tigecycline. The role of porins is not only limited to resistance, as porins are membrane-stability contributors and are involved in various physiological and immunological processes. Furthermore, specific porins, such as Omp35 and Omp36, have the potential to play a role in the efflux of antibiotics from the periplasmatic space to the external environment (Taherpour and Hashemi, 2013).

## Regulators

Molecular regulators constitute one of the most important components influencing antimicrobial resistance in *Enterobacterales*. They serve as key inducers capable of modulating the membrane permeability by regulating essential elements such as membrane transporters, porins and efflux pumps. There are many regulators, such as *ramA*, *marA*, *soxS*, and *robA* (Davin-Regli et al., 2019).

*RamA* was first detected in *Enterobacter*, and is responsible for conferring resistance to various antibiotic drugs, like tetracycline, tigecycline, and fluoroquinolones. The regulatory mechanism of *ramA*-induced resistance involves reducing the expression of Omp35 and promoting an active efflux mechanism. In *Enterobacter* species, *ramA* is a regulator of membrane permeability, working independently of the *marA* regulator. Moreover, *rarA* is linked with the regulation of the *ramA*-*marA* cascade. *RamA* regulator was well characterized in *Enterobacter*, *Klebsiella* and *Salmonella* (Davin-Regli et al., 2019). In *Enterobacter cloacae ramA* is associated with overexpression of the efflux pumps, such as the AcrAB efflux pump. Additionally, overexpression of *acrB* and *ramA* is considered a major factor contributing to tigecycline resistance in *Enterobacter cloacae* species, but further investigations need to be done (Chen et al., 2017; Jiang et al., 2022). Moreover, other studies documented activation of AcrAB-TolC and OqxAB efflux pumps by other regulators, such as *marA*, *soxS*, *robA*, or *acrR*. This observation indicates that *ramA* is not always a necessary regulator, as *marA* can also elicit overexpression of the AcrAB pump, for instance in *Klebsiella* species (Goodarzi et al., 2021).

In summary, certain transcriptional regulators, as mentioned previously, have the ability to upregulate the expression of RND superfamily efflux pumps, contributing to the emergence of tigecycline resistance, and frequently and subsequently the MDR phenotype.

## Antibiotic tolerance

Recent studies have shown an interesting phenomenon of bacterial adaptation to different antibiotic treatments. This mentioned phenomenon is called antibiotic tolerance and it is associated with repetitive selective pressure via treatments with antimicrobial agents. Moreover, it is suspected that the bacterial subpopulations would become tolerant and resistant to the drug used. Antibiotic tolerance can be confused with heteroresistance, which differs from antibiotic tolerance by instability of population. A tolerant population shows no difference in MIC and can survive higher concentrations of antibiotics than the MIC. However, the subpopulation can survive, and the bacterial cells cannot replicate during antibiotic treatment (Brauner et al., 2016; Sulaiman and Lam, 2021).

Bacteria have a remarkable ability to adapt to different environmental conditions. In response to antibiotic pressure subpopulations with advantageous traits can survive. These cells may acquire genetic mutations that confer tolerance or increased spore formation. There is a possibility of increasing a population’s tolerance by applying similar stresses. Moreover, the population can exhibit different physiology compared to the original population. The development of mutations can be an effect of antibiotic treatment or can occur spontaneously. Consequently, those tolerant groups have the potential to dominate the population through repetitive antibiotic treatments (Sulaiman and Lam, 2021).

The study performed by Fridman et al. showed the ability of *E. coli*, representative of *Enterobacterales* to become drug-tolerant (Fridman et al., 2014). Overnight incubation in an ampicillin-containing medium exhibited an increase in tolerance over time with extended lag time. Lag time is a period in the bacterial cells’ life when they are adjusting to a new environment before their growth. Moreover, these population could adjust their lag time to the duration of antibiotic exposure, suggesting an optimization of lag time based on previous treatments through their mutations. During research, eight mutations were identified. The mutated genes included genes coding specific enzymes and cellular components. The exact mechanism leading to the extension of lag time is still unknown (Sulaiman and Lam, 2021).

It has been observed that tolerance frequently emerges before resistance in the evolutionary process. This phenomenon is explained by a higher frequency of tolerance mutations than resistance mutations, possibly due to the larger target size. Screening for development of tolerance is also needed to reduce the rate of resistance. Constant monitoring of the pathogen’s tolerance is crucial when patients receive antibiotics. If tolerance is detected, drugs or treatment need to be changed. Tolerance mutations can be antibiotic specific, and switching of the drug could influence the positive effects of treatment (Fridman et al., 2014; Sulaiman and Lam, 2020; Sulaiman and Lam, 2021).

## Concluding remarks

The emergence and spread of antibiotic resistance among *Enterobacterales* poses a significant threat to human health. Tigecycline a representative of glycylcyclines is considered a last-resort therapeutic option for MDR infections, especially when microorganisms are already resistant to carbapenems and/or colistin. Nevertheless, increasing cases of tigecycline resistance in *Enterobacterales* strains causing concerns about the effectiveness of this antimicrobial agent. Major mechanisms of tigecycline resistance include efflux pumps, the *tet* genes family and others, such as heavy metals, OM porins and systems of regulators. Another interesting fact is the wide dissemination of ARGs via a process called HGT, resulting in the spread of genes within the same species and across different groups of microorganisms. Various antimicrobial agents are used widely, not only in hospital settings. Other environments, where antibiotics and antibiotic resistance genes can occur are animals (pigs, ducks, cows and geese) and their faeces, soil, water, and wastewater. A study performed in 2013 showed that almost one hundred thousand tons of antibiotics were consumed in China. Antibiotic-resistant genes exhibited rapid development due to misuse and overuse of antimicrobial agents. Moreover, there were cases of prescribing antibiotics to patients with seasonal influenza. Due to alarming reports compiled by CDC/WHO, a great effort needs to be taken to reduce the use of antibiotics and restrict them only to major cases (Qiao et al., 2018).

## Author contributions

LK: Conceptualization, Data curation, Formal analysis, Investigation, Methodology, Visualization, Writing – original draft. PiM: Conceptualization, Data curation, Funding acquisition, Investigation, Supervision, Validation, Writing – review & editing. DI: Investigation, Methodology, Writing – original draft. PS: Data curation, Formal analysis, Writing – original draft. MM: Formal analysis, Methodology, Writing – original draft. PW: Data curation, Formal analysis, Writing – original draft. PaM: Validation, Visualization, Writing – review & editing. AW: Formal analysis, Resources, Writing – review & editing. PR: Formal analysis, Supervision, Validation, Writing – review & editing. ET: Conceptualization, Supervision, Validation, Writing – review & editing.
